# Switching Pharmacological Treatment in Wilson Disease: Case Report and Recommendations

**DOI:** 10.1177/2324709619896876

**Published:** 2020-01-10

**Authors:** Marcia Leung, Jaimie Wu Lanzafame, Valentina Medici

**Affiliations:** 1 University of California Davis, Sacramento, CA, USA

**Keywords:** Wilson disease, penicillamine, zinc acetate, trientine

## Abstract

*Background*. Available treatments for Wilson disease (WD) prevent longterm complications of copper accumulation. Current anti-copper agents include zinc salts, penicillamine, and trientine. Patients with WD may switch between the agents for a number of reasons. Due to the different mechanisms of action between the copper chelators and zinc salts, transitioning could require a period of overlap and increased monitoring. There are no large studies that investigate the best transition strategies between agents. In this article, we review the treatments for WD and how to monitor for treatment efficacy. *Case Summary*. The patient had been diagnosed with WD for over 20 years prior to establishing care in our Hepatology Clinic. During his initial course, he was transitioned from penicillamine to zinc due to evidence suggesting penicillamine had greater adverse effects in the long term. Later, he was switched to trientine. His liver enzymes and 24-hour urine copper were monitored. During these years, he intermittently had some financial hardship, requiring him to be on penicillamine rather than trientine. He also had developed acute kidney injury. Overall, his liver disease remained under control and he never had signs of decompensated cirrhosis, but had fluctuations of liver enzymes over the years. *Conclusion*. Anti-copper treatment for WD has to be tailored to medication side effects profile, patient’s chronic and emerging comorbidities, as well as costs. Transitioning regimens is often challenging, and it requires closer monitoring, with no predictors of response.

## Introduction

Wilson disease (WD) is an autosomal recessive disorder with a prevalence traditionally reported of 1 in 30,000 individuals,^1,2^ but more recent studies indicate that it could affect 1 in 7,026 individuals.^3,4^ The underlying genetic defect is based on disease-causing mutations in the *ATP7B* gene,^5^ which encodes a copper transporting adenosine triphosphatase crucial for biliary copper excretion as well as synthesis and maturation of functional ceruloplasmin.^6,7^ Absent or reduced function of this protein results in copper accumulation within multiple organs, most notably the liver, brain, and cornea. While patients may present with a variety of symptoms, the clinical manifestations of WD are predominately hepatic, neurologic, and psychiatric. There are limited medical treatment options for WD that have shown to prevent sequelae of the disease and little research has been done to determine the most effective way to transition between agents in the event of ineffective therapy or a patient’s inability to tolerate the medication.

## Case Presentation

The patient was first diagnosed with WD at age 32, when he presented with elevated liver enzymes and workup revealing low ceruloplasmin. He started to be followed at University of California Davis Hepatology Clinic when he was 56 years old. His comorbidities include well-controlled HIV on efavirenz/emtricitabine/tenofovir.

At the time of establishing care at University of California Davis in 2006, he had compensated cirrhosis with elevated liver enzymes and mild hepatomegaly. He underwent liver biopsy, which demonstrated moderate portal and lobular lymphocytic infiltrate, periportal and focal bridging fibrosis (stage 2-3), and moderate steatosis (25%). Drug-induced liver injury was considered, especially with ongoing highly active antiretroviral therapy, as approximately 20% of all patients on highly active antiretroviral therapy have liver enzymes abnormalities.^8^ Other causes of elevated liver enzymes including alcohol abuse, viral hepatitis, autoimmune hepatitis, α-1 antitrypsin deficiency, and hemochromatosis were excluded. He had been on penicillamine (PCA) 250 mg 3 times daily for more than 20 years, and his HIV was under control as shown by CD4 count 543 cells/mm^3^ and viral load <50 copies/mL. His past medical history includes nephrolithiasis, major depressive disorder, hypertension, and type 2 diabetes.

On physical examination, the patient had no scleral icterus. Abdomen nondistended, liver palpable at about 1 cm under the right costal margin with sharp edge. No collateral veins, ascites, peripheral edema, flapping tremor, signs of encephalopathy, and spider angiomata. No focal neurologic deficits on physical examination, cranial nerves intact, alert, and oriented to person, place, time, and situation.

### Laboratory

Negative serology for hepatitis B (positive HBsAb) and C. chemistry panel showed normal electrolytes and kidney function (sodium 145 mEq/L, potassium 4.1 mEq/L, chloride 105 mEq/L, bicarbonate 26 mEq/L, blood urea nitrogen 11 mg/dL, creatinine 0.6 mg/dL, and calcium 9.4 mg/dL). Bilirubin levels were 0.7 mg/dL, alkaline phosphatase 96 IU/L, aspartate transaminase 54 IU/L, and alanine transaminase 84 IU/L. Blood count was within normal limits including normal platelets.

### Imaging

Ultrasound of abdomen demonstrated hepatomegaly with moderate degree of hepatosteatosis with evidence of portal hypertension with splenomegaly, and no ascites. Magnetic resonance imaging of brain demonstrated mild cerebral cortical volume loss. There was no evidence of alterations in the striatum or in the white matter.

## Final Diagnosis, Treatment, Outcome, and Follow-up

The patient was compliant on PCA for more than 20 years when he established care in 2007, but his transaminases were persistently elevated. Compliance was confirmed by 24-hour urinary copper levels in 300 to 400 µg range. Given growing evidence that PCA treatment is affected by several long-term side effects,^9^ including nephrotoxicity with proteinuria and hematuria, skin progeric changes, and autoimmune conditions,^10^ he was switched over to zinc acetate shortly after establishing care.^6^

**Figure 1.**
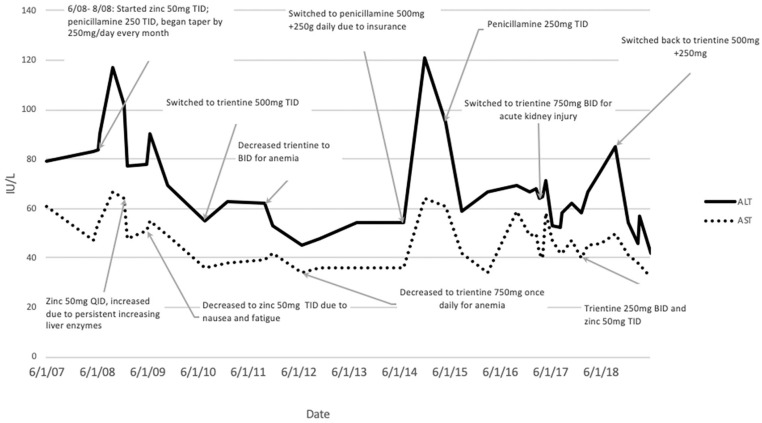
ALT and AST levels trends. Abbreviations: ALT, alanine transaminase; AST, aspartate transaminase; BID, 2 times a day; TID, 3 times a day; QID, 4 times a day.

**Figure 2.**
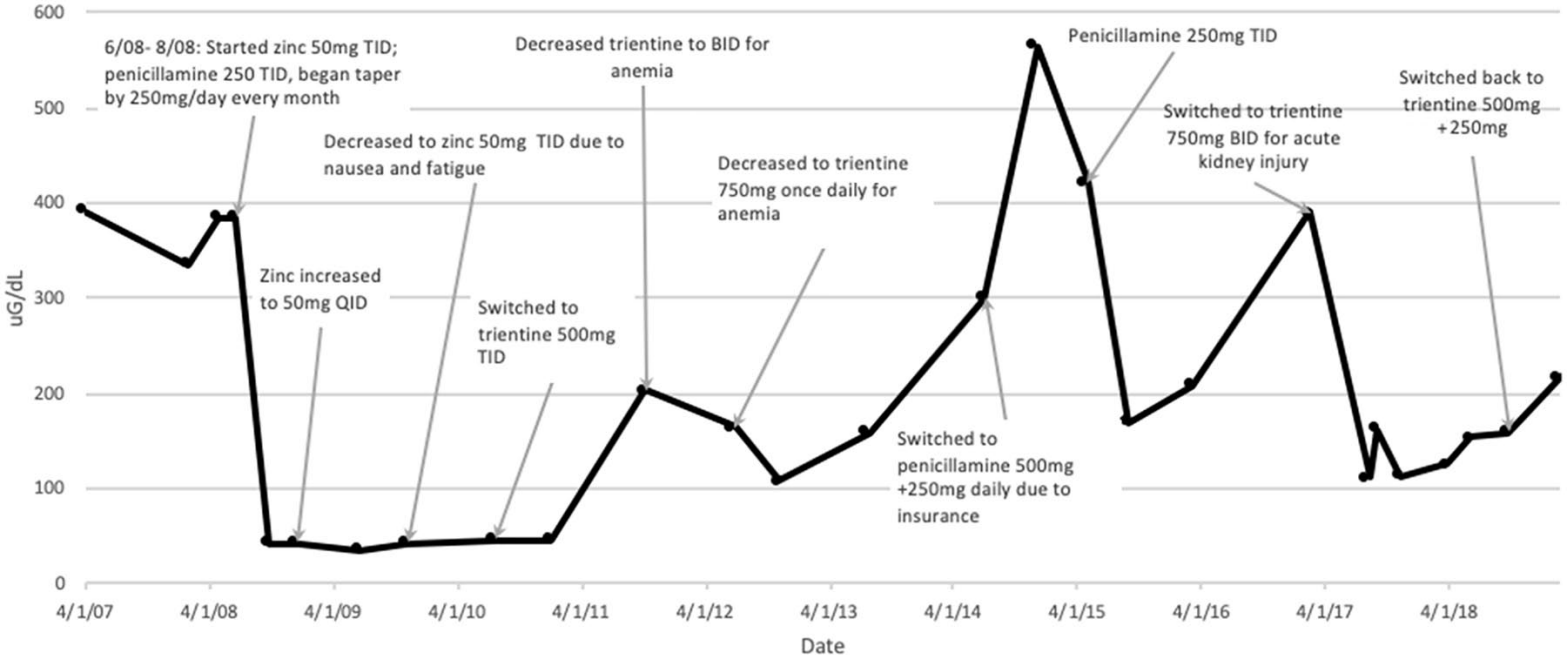
Twenty-four-hour urine copper trends. Abbreviations: BID, 2 times a day; TID, 3 times a day; QID, 4 times a day.

In July 2010, given persistent elevation of liver enzymes (aspartate transaminase 55-90 IU/L, alanine transaminase 36-55 IU/L) and new published evidence that chelation treatment had lower rates of treatment failure and orthotopic liver transplantation, he was switched to trientine (Table 1).^9^ From 2011 to 2014, dose adjustments to trientine were made (Figures 1 and 2) mainly due to development of mild normocytic anemia, hemoglobin of 11.5 g/dL from baseline of 15 g/dL, with mean corpuscular volume of 89 fL. This was attributed to overtreatment with trientine as other causes of anemia were excluded, and there were no changes to HIV medications. In 2015, he eventually had to stop trientine due to lack of insurance coverage and was switched to PCA.

**Table 1.** Transitioning Between Therapeutic Agents in Wilson Disease^a^.

	When to Transition	How to Transition	Monitoring
PCA to trientine	● Patient unable to tolerate PCA ● Development of nephrotic syndrome, severe thrombocytopenia, or aplastic anemia	No taper or overlap indicated	Baseline CBC, CMP, and 24-hour urinary copper prior to switch Repeat above laboratory tests monthly for 3 to 4 months *Goal*: Maintain 24-hour urinary copper 200 to 500 µg, stable ALT *Long-term*: Repeat blood tests, including CBC and CMP, and 24-hour urinary copper at least every 6 months; 24-hour urinary copper should be in the 200 to 500 µg/day range
PCA to zinc salts	● Patient unable to tolerate PCA ● Development of renal failure, severe thrombocytopenia/aplastic anemia ● Worsening neurologic symptoms ● Pregnancy	Start zinc at 50 mg TID, uptitrate by 50 mg increments as necessary Continue PCA for at least 3 months after initiating zinc therapy PCA and zinc dosing must be spread out so that they are not taken at the same time; PCA cannot be given at meal times	CMP, 24-hour urinary copper prior to switch and every 3 months until urinary copper at goal/stabilizes *Goal*: Maintain urinary copper <75 µg, stable liver enzymes *Long-term*: Repeat blood tests, including CBC and CMP, and 24-hour urinary copper at least every 6 months; 24-hour urinary copper should be <75 µg/day
Trientine to zinc salts	● Financial limitations ● Limited drug availability ● Development of pancolitis ● Pregnancy	Start zinc at 50 mg TID, titrate by 50 mg increments as necessary; when starting zinc, reduce trientine dose by 250 mg and reduce by 250 mg every month until termination of trientine Continue trientine for at least 3 months after initiating zinc therapy Trientine and zinc dosing must be spread out so that they are not taken at the same time; PCA cannot be given at meal times	CMP, 24-hour urinary copper prior to switch and every 3 months until urinary copper at goal/stabilizes *Goal*: Maintain urinary copper <75 µg, stable liver enzymes *Long-term*: repeat blood tests, including CBC and CMP, and 24-hour urinary copper at least every 6 months; 24-hour urinary copper should be <75 µg/day
Zinc salts to trientine	● Ineffective therapy demonstrated by uptrending liver enzymes, development of liver synthetic dysfunction	No taper or overlap indicated	Baseline CBC, CMP, and 24-hour urinary copper prior to switch Repeat above laboratory tests monthly for 3 to 4 months *Goal*: Maintain urinary copper 200 to 500 µg, stable liver enzymes
Trientine or zinc salts to PCA	● Financial limitations ● Patient’s preference	No taper or overlap indicated	Baseline CBC, CMP, and 24-hour urinary copper prior to switch Repeat above laboratory tests monthly for 1 to 2 months *Goal*: Maintain urinary copper 200 to 500 µg, stable liver enzymes *Long-term*: Repeat blood tests, including CBC and CMP, and 24-hour urinary copper at least every 6 months; 24-hour urinary copper should be in the 200 to 500 µg/day range Repeat UA every 6 months to check on proteinuria

Abbreviations: PCA, penicillamine; CBC, complete blood count; CMP, complete metabolic panel (including liver enzymes); ALT, alanine transaminase; TID, 3 times a day; UA, urinalysis.

a Dose of zinc refers to elemental zinc.

In February 2017, he developed acute kidney injury (elevation in baseline creatinine from 0.84 to 1.34 mg/dL) following a unilateral renal stone, for which he underwent cystoscopy with laser lithotripsy and stent placement. He was also noted to have proteinuria 50 mg/dL following this incident, so in an attempt to minimize contributing nephrotoxic agents, he was switched off of PCA to trientine. His antiretroviral therapy for HIV was also changed from efavirenz/emtricitabine/tenofovir to emtricitabine/rilpivirine/tenofovir alafenamide. As a result, his creatinine downtrended to baseline by June 2017. Liver enzymes were stable during this time with no acute rises.

In March 2018, new financial issues prevented him to continue with the full dose of copper chelating agent. At that point, zinc acetate was added to trientine. In October 2018, he was back on trientine alone again as he obtained full insurance coverage while transaminases remained normal.

## Discussion

Therapies for WD include chelating agents, PCA, and trientine, which increase urinary copper excretion and zinc salts that inhibit intestinal copper absorption. The goal of initial treatment of WD is to reestablish copper balance, whereas the maintenance phase serves to maintain these levels without inducing copper deficiency.^6^ Maintenance represents lifelong therapy, unless a patient undergoes liver transplant.^11^ However, despite its side effects and limitations, current medical treatment for WD is effective in avoiding disease progression and liver transplant for the majority of patients provided treatment is tailored and optimized to the patient’s needs and comorbidities. In particular, we are not aware of case reports describing WD treatment options and outcomes in patients with HIV. Therefore, connections between the available literature and the current case may appear marginal and based on the treating clinician experience and assessment.

Penicillamine was among the first oral agents introduced for treating WD.^12^ Although multiple studies have demonstrated its efficacy in treatment of WD,^13-16^ PCA has an extensive side effect profile. Patients can experience early hypersensitivity reactions within 1 to 3 weeks of initiation of treatment, characterized by fever, cutaneous eruptions, cytopenias, and proteinuria. Late-onset reactions, although rare, may be fatal and include drug-induced lupus, nephrotic syndrome leading to renal failure, severe thrombocytopenia, or aplastic anemia.^13,17-19^ In the event of a severe reaction, PCA should be discontinued and patient should be transitioned to alternative therapy.^19^ PCA should be avoided in patients with a penicillin allergy, history of PCA-related aplastic anemia or agranulocytosis, or renal disease. When possible, it should also be avoided in patients with neuropsychiatric manifestations of WD, as at least 10% of those treated have had worsening of symptoms.^8,20-22^ In the described case, the rationale for initially switching from PCA to zinc acetate was the concern about emerging of autoimmune conditions and nephropathy in a patient with multiple comorbidities and on lifelong pharmacological treatment.

Trientine was developed as a therapeutic option for patients who were unable to tolerate or had contraindications to PCA. It has been shown to be as effective for treatment of WD and associated with fewer side effects compared with PCA.^23-26^ Although hypersensitivity reactions and pancytopenia can occur with trientine, these side effects are rare and can often be addressed with dose reduction and use of steroids. While worsening of neuropsychiatric symptoms has also been noted with trientine use, its incidence is lower.^6,18,27^ Rare side effects of trientine include sideroblastic anemia, pancolitis, and hemorrhagic gastritis. Notably, a large barrier to use of trientine therapy is availability or cost of the drug, as seen in our patient described above.

The main advantage of zinc salts over chelating agents is that they are generally well tolerated by patients due to fewer side effects. Gastric irritation is the most commonly described side effect, though that may be due to the salt preparation rather than zinc itself. Unlike the chelating agents, zinc can also be safely given during pregnancy without dose reduction. Zinc has been shown to be effective in controlling copper levels during maintenance therapy,^15,28-30^ though in some studies, chelators have been shown to be better at slowing progression of WD if tolerated.^9,31^ In our patient, despite compliance with zinc therapy, he had rising liver function tests, suggesting ineffective maintenance therapy with zinc. It has also been suggested that zinc may be as effective as chelators as initial therapy,^15,32-34^ especially in patients who are unable to tolerate chelating agents, who have significant neuropsychiatric disease, or in patients who are presymptomatic.

Regardless of therapy selection, patients must be closely monitored clinically and with routine laboratory tests while on therapy. With initiation of treatment or modification of regimen, patients should be monitored at least every 3 months for efficacy of treatment, compliance, and potential therapy adverse effects. In addition to clinical evaluation, recommended laboratory tests included liver enzymes, international normalized ratio, complete blood count, urinalysis, and surrogates of copper metabolism, including serum-free copper, ceruloplasmin, and 24-hour urinary copper excretion. Monitoring of urine copper may be the most effective way to evaluate compliance and success of therapy.^6,35^ When on chelating agents, elevated values of urine copper may suggest non-adherence and hepatic deterioration, whereas low values may indicate overtreatment when accompanied by low non–ceruloplasmin-bound copper.^6,36^ Conversely, if a patient is on zinc, marked reduction of urine copper represents reduction of total body copper, implying effective therapy with zinc. Monitoring liver enzymes also provides insight into efficacy of therapy. Rising liver enzymes despite compliance to therapy may suggest ineffective treatment and the need to transition to an alternative agent.

While several studies have been done regarding the efficacy of chelating agents^13^ and zinc, as well as comparing the various agents available for treatment of WD,^14,15,34,37^ there is little to no commentary on dose adjustments or the most effective way to transition between different agents if necessary. As demonstrated by our patient above, patients may be transitioned between therapies for various reasons, whether it be inability to tolerate therapy due to adverse effects, ineffective treatment, comorbidities, or financial concerns. Since zinc typically requires 2 to 6 months of treatment during initial therapy to reach the maintenance phase, tapering is likely required when switching to zinc. Transitioning between agents is further complicated when on multiple agents due to timing of medications as absorption of chelators is affected by meals.^6,18^ In addition, clinicians can prescribe other agents including vitamin E, which may be beneficial given its antioxidant properties and some limited evidence of reduced levels of vitamin E in plasma of patients with WD.^38^ It is also noticeable that other treatment options may become soon available for the treatment of WD, and they include tetrathiomolybdate bis-choline^39^ and methanobactin.^40^ An additional layer of complexity is related to the possible combination treatment. Even though most clinicians are concerned about the binding of zinc by copper chelators if administered concomitantly, data from Askari at al^41^ showed that the combination treatment with trientine and zinc in case of decompensated cirrhosis is associated with significant improvement of liver function. As shown in Table 1, we are proposing specific recommendations for transitioning between different therapeutic agents.

In conclusion, although there have been studies exploring the efficacy of various treatments for WD and comparing these agents, there is limited research regarding effective transitions between these agents. We have proposed specific recommendations for transitioning between therapeutic agents. However, further studies are warranted to help determine the optimal transition indications, timing, and monitoring.
